# Development of a predictive model of hospitalization in primary care patients with heart failure

**DOI:** 10.1371/journal.pone.0221434

**Published:** 2019-08-16

**Authors:** Luis García-Olmos, Río Aguilar, David Lora, Montse Carmona, Angel Alberquilla, Rebeca García-Caballero, Luis Sánchez-Gómez, the CHIC Group

**Affiliations:** 1 Multiprofessional Education Unit for Family and Community Care (South-east), Madrid, Spain; 2 Health Service Research Network for Chronic Diseases (*Red de Investigación en Servicios de Salud en Enfermedades Crónicas/REDISSEC*), Madrid, Spain; 3 Cardiology Department, La Princesa University Teaching Hospital, Madrid, Spain; 4 Clinical Research Unit (imas12-CIBERESP), Hospital Universitario 12 de Octubre, Madrid, Spain; 5 Agency for Health Technology Assessment, Carlos III Institute of Health (*Instituto de Salud Carlos III/ISCIII*), Madrid, Spain; 6 Multiprofessional Education Unit for Family and Community Care (Centre), Madrid, Spain; 7 Internal Medicine Department Infanta Sofía University Teaching Hospital, Madrid, Spain; Universita degli Studi di Napoli Federico II, ITALY

## Abstract

**Background:**

Heart failure (HF) is the leading cause of hospitalization in people over age 65. Predictive hospital admission models have been developed to help reduce the number of these patients.

**Aim:**

To develop and internally validate a model to predict hospital admission in one-year for any non-programmed cause in heart failure patients receiving primary care treatment.

**Design and setting:**

Cohort study, prospective. Patients treated in family medicine clinics.

**Methods:**

Logistic regression analysis was used to estimate the association between the predictors and the outcome, i.e. unplanned hospitalization over a 12-month period. The predictive model was built in several steps. The initial examination included a set of 31 predictors. Bootstrapping was used for internal validation.

**Results:**

The study included 251 patients, 64 (25.5%) of whom were admitted to hospital for some unplanned cause over the 12 months following their date of inclusion in the study. Four predictive variables of hospitalization were identified: NYHA class III-IV, OR (95% CI) 2.46 (1.23–4.91); diabetes OR (95% CI) 1.94 (1.05–3.58); COPD OR (95% CI) 3.17 (1.45–6.94); MLHFQ Emotional OR (95% CI) 1.07 (1.02–1.12). AUC 0.723; R2N 0.17; Hosmer-Lemeshow 0.815. Internal validation AUC 0.706.; R2N 0.134

**Conclusion:**

This is a simple model to predict hospitalization over a 12-month period based on four variables: NYHA functional class, diabetes, COPD and the emotional dimension of the MLHFQ scale. It has an acceptable discriminative capacity enabling the identification of patients at risk of hospitalization.

## Introduction

Heart failure (HF) is a chronic clinical syndrome that affects more than 2% of the population. The number of HF patients is expected to rise significantly in coming years [1]. These patients have a high mortality rate and hospitalization is frequent.

Hospital admission rates for HF patients have been consistently high in recent decades. HF is the number one cause of hospital admission for patients over 65. Care of HF patients consumes 2% of the health budget and over 70% of the expense is due to the hospital care received by these patients [2].

Predictive models to identify risk factors for hospital readmission have been developed to prevent the re-hospitalization of these patients [3–6]. These studies have become more frequent over the last 10 years and are most prevalent in the United States, some featuring data from clinical trials, others using data from administrative databases and most focusing on hospital populations, including patients who have been hospitalized for HF.

The patients included in clinical trials are different from HF patients in the community [7]. While in the community, more than 80% of HF patients fall into NYHA functional class I-II, but patients in functional class III-IV are included in clinical trials. Omissions and registration errors are frequent in studies that use administrative databases.

Different types of factors have been studied as possible causes of hospitalization: sociodemographic, clinical, psychosocial and health system. A high number of risk factors for the hospitalization have been identified [8] but results are inconsistent. Clinical variables have been the focus of most studies but in the case of hospitalization they have a low—mild explanatory capacity. The discrimination capacity, measured by the C-statistic, is in the 0.6 to 0.8 range.

Most studies use 30 days as the reference period for rehospitalization, some have a 180-day reference period and only a few seek to predict hospitalization within a year’s time. HF rehospitalization studies with short periods such as 30 days, mainly analyse the quality of hospital care received by HF patients, while studies with rehospitalization reference periods of one year assess continuity of care and the complete healthcare process.

Most studies analyse readmissions related to HF. However, in population studies, HF-related hospitalization only accounts for 16.5% of the cases; 21% are due to other cardiovascular diseases, and in more than 60% of the cases they are due to non-cardiovascular causes [9].

Considering that HF patients are complex and have a high comorbidity rate [10], an analysis of hospital admissions for all causes over a long period can help to understand the role played by comorbidity and the entire healthcare process, including continuity, in the admission rates of these patients.

The aim of this study is to develop and internally validate a predictive model for hospital admission in a year’s time for any non-programmed cause, in patients with heart failure receiving primary care.

## Methods

This is part of a broader study whose methodology has been previously described [11]. This is a prospective cohort study conducted in two cities of the Community of Madrid which together have a population of 132 851. The study includes all patients over 18 diagnosed with HF, treated at the seven health centres that exist in the two municipalities.

### Inclusion criteria

1) patients who meet the Framingham criteria for HF diagnosis; 2) left ventricular ejection fraction (LVEF) less than 50% or significant structural lesion and/or diastolic dysfunction; patients who met the Framingham criteria and who had an echocardiographic study performed in the six months prior to inclusion were also included, and 3) informed consent of the patient to participate in the study.

### Exclusion criteria

1) institutionalized patients; 2) patients with a terminal illness other than HF; and 3) patients with a life expectancy of under 6 months.

### Determinations

a) Dependent variable: Unplanned hospitalization in the 12 months following inclusion in the study; b) Predictive variables: 1) disability, measured using version 2 of the 36-item World Health Organization Disability Assessment questionnaire (WHODAS-2) [12], obtaining a global score and a score in each of the six domains covered by the questionnaire: understanding and communication (UCA), getting around (GAR), self-care (SCA), getting along with people (GAP), life activities (LAC), and participation in society (PSO); 2) quality of life, measured by the Minnesota Living with Heart Failure Questionnaire (MLHFQ), obtaining a global score and a score in each of the two domains included in the questionnaire: physical and emotional [13]; 3) In addition to the questionnaire scores, the following variables were also analysed: age, sex, marital status, living alone, work situation, educational level, NYHA functional status, left ventricular ejection fraction (LVEF), amount of time since heart failure was first diagnosed, body mass index (BMI), blood pressure, use of drugs, comorbidity and hospitalization for any unplanned reason in the year prior to inclusion.

For the purposes of the multivariate analysis, WHODAS-2 scores were recoded into two categories: mild / moderate disability v. severe / extreme disability. NYHA functional class was recoded into two categories: class I-II and class III-IV. Educational level was divided into two categories: low (compulsory education, up to age 16) and medium / high (vocational training and university studies).

The initial evaluation consisted of a consultation with the physician and another with the nurse. In the visit with the general practitioner: the GP verified whether patients had an ECHO performed in the previous six months, assessed their baseline situation, checked inclusion and exclusion criteria, Framingham criteria, NYHA functional class and drug treatment; an echocardiogram and proBNP determination were also requested, where necessary. Nursing staff assessed patients’ functional status (WHODAS-2) and quality of life (MLHFQ)

To establish predictive models based initially on 31 variables (not all included in the final model), a sample of 250 patients was considered sufficient, with an estimated hospitalization rate of 24% in the first 12 months [14,15].

### Development phase of the predictive model

Logistic regression analysis was used to estimate the correlation between predictors and outcome, unplanned hospitalization in 12 months. The predictive model was built in several steps. 1) The initial examination included a set of 31 predictors (Table 1). The relationship between a predictor and admission to hospital was estimated using a crude odds ratio, area under a receiver operating characteristic (AUROC) curve and R^2^. 2) The full model with 31 predictors was developed using penalized maximum likelihood estimation to directly correct the model for over-optimism. It was further simplified by decreasing the number of predictors based on recommendations found in the literature [16]. Interaction analysis was performed but no significant interaction was found. 3) Discrimination of this final model was quantified via an AUROC curve. Discrimination describes the ability of the prognostic model to distinguish patients with the outcome from those without the outcome. Predictive ability was determined using Nagelkerke's R^2^ and the Brier score index. Nagelkerke’s R^2^ is the amount of variability in outcomes that is explained by the prediction model while the Brier score is a performance measurement quantifying the gap between observed and predicted outcome. Lastly, calibration was tested with the Hosmer-Lemeshow test for goodness of fit. Statistical analyses were performed using the R Regression Modeling Strategies package version 3.6–3.

**Table 1:** Description of predictor variables.

	Overall	Not Hospitalized	Hospitalized	p-value
n	251	187	64	
No EDC CHRONIC (mean (sd))	7.40 (2.85)	7.32 (2.86)	7.65 (2.84)	0.423
TIME_EVOLUTION (mean (sd))	4.89 (6.00)	5.00 (6.38)	4.55 (4.77)	0.608
PREVIOUS HOSPITALIZATION %	92 (36.8)	62 (33.3)	30 (46.9)	0.074
AGE (mean (sd))	74.86 (9.97)	74.18 (10.01)	76.86 (9.64)	0.063
WOMEN (%)	139 (55.4)	101 (54.0)	38 (59.4)	0.549
NYHA III—IV (%)	51 (20.3)	29 (15.5)	22 (34.4)	0.002
LIVE ALONE (%)	52 (20.7)	37 (19.8)	15 (23.4)	0.657
MEDIUM / HIGH EDUCATIONAL LEVEL (%)	37 (14.7)	31 (16.6)	6 (9.4)	0.231
EMPLOYMENT SITUATION (%)				0.668
1	93 (37.1)	69 (36.9)	24 (37.5)	
2	140 (55.8)	103 (55.1)	37 (57.8)	
3	18 (7.2)	15 (8.0)	3 (4.7)	
BMI (mean (sd))	30.88 (6.12)	30.98 (5.86)	30.58 (6.87)	0.659
DIABETES	100 (39.8)	67 (35.8)	33 (51.6)	0.038
COPD (%)	35 (13.9)	19 (10.2)	16 (25.0)	0.006
CORONARY HEART DISEASE %	62 (24.7)	47 (25.1)	15 (23.4)	0.917
ATRIAL FIBRILLATION (%)	141 (56.2)	101 (54.0)	40 (662.5)	0.300
STROKE (%)	43 (17.1)	28 (15.0)	15 (23.4)	0.174
LVEF (mean (sd))	58.04 (12.38)	57.04 (12.35)	61.06 (12.12)	0.038
ACE/ARB = 1 (%)	177 (70.5)	134 (71.7)	43 (67.2)	0.604
BETA BLOQUERS (%)	142 (56.6)	111 (59.4)	31 (48.4)	0.169
DIURETIC (%)	208 (82.9)	153 (81.8)	55 (85.9)	0.574
CALCIUM CHANNEL BLOCKERS (%)	65 (25.9)	46 (24.6)	19 (29.7)	0.524
DIGOXIN (%)	65 (25.9)	46 (24.6)	19 (29.7)	0.524
PHYSICAL MLHFQ (mean (sd))	16.24 (10.24)	15.26 (10.06)	19.09 (10.04)	0.010
EMOTIONAL MLHFQ (mean (sd))	6.41 (6.26)	5.61 (5.88)	8.73 (6.79)	0.001
TOTAL MLHFQ (mean (sd))	24.25 (16.47)	22.20 (15.64)	30.25 (17.47)	0.001
UCA WHODAS SEVERE / EXTREME (%)	33 (13.2)	21 (11.3)	12 (18.8)	0191
GAR WHODAS SEVERE / EXTREME (%)	93 (37.2)	63 (33.7)	30 (47.6)	0.068
SCA WHODAS SEVERE / EXTREME (%)	36 (14.4)	20 (10.8)	16 (25.0)	0.009
GAP WHODAS SEVERE / EXTREME (%)	28 (11.2)	18 (9.7)	10 (15.6)	0.290
LAC WHODAS SEVERE / EXTREME (%)	101 (40.4)	64 (34.4)	37 (57.8)	0.002
PSO WHODAS SEVERE / EXTREME (%)	61 (24.4)	34 (18.3)	27 (42.2)	0.001
TOTAL WHODAS SEVERE / EXTREME (%)	43 (17.1)	26 (13.9)	17 (26.6)	0.033

EDC: Expanded diagnostic clusters. NYHA: New York Heart Association. Body Mass Index. LVEF: Left Ventricular Ejection Fraction. ACE/ARB: Ratio ACE Inhibitors /Angiotensin Receptor Blockers. MLHFQ: Minnesota Living with Heart Failure Questionnaire. WHODAS: World Health Organization Assessment Schedule. UAC: Understanding and Communication. GAR: Getting around. SCA: Self-Care. GAP: Getting along with people. LAC: Life activities. PSO: Participation in society

### Internal validation phase

Internal validation was evaluated using the bootstrapping technique, simulating 1 000 samples with 251 subjects similar to the original sample. The predictive ability of the model was evaluated internally based on discrimination (AUROC curve), measures of overall performance (Nagelkerke's R^2^ and the Brier score index) and calibration [17]. Calibration of the model was assessed graphically and estimated with the calibration intercept and slope. In case of a perfect fit between the model and the data, the calibration intercept is equal to 0 and slope is equal to 1.

The study was approved by the Clinical Research Ethics Committee of La Princesa University Teaching Hospital, and informed consent in writing was obtained from all patients before being enrolled.

## Results

The performance measures of predictive model used to establish the validity and utility were AUROC curve, Nagelkerke's R2 and the Brier score index. Robustness of performance measures of modes were evaluated using internal validation using bootstrap method.

278 patients were recruited, 27 of whom were excluded for the following reasons: 21 because after consulting the physician they left without meeting with the nurse, 1 due to CRD registration errors and 5 patients because at the time of this analysis, 12 months had not yet gone by since the date of their inclusion. The excluded patients were similar to those analysed in terms of age, sex, and NYHA functional class.

Of the 251 patients included in the study, 64 (25.5%) were admitted to hospital for some unplanned reason in the 12 months following the date of inclusion. The baseline characteristics of these patients are shown in Table 1. The mean age was 74.86 ± 9.97, 55.4% were women, 20.3% of the patients were in NYHA functional class III-IV and the mean LVEF was 58.04 ± 12.35.

Table 2 shows the results of the univariate analysis of the predictor variables included in the study. The effect is expressed in terms of OR, with its confidence intervals and explanatory participation in the occurrence of the event, expressed by the R2N and in the discriminative capacity, expressed by the AUC. NYHA functional class, diabetes, COPD, LVEF, and the variables that measure quality of life and disability are the ones found to have a statistically significant effect on hospitalization.

**Table 2:** Univariate analysis.

	OR	OR 95% CI	p-value	R2N	ROC area
No EDC CHRONIC	1.04	0.94–1.15	0.4219	0.004	0.534
TIME_EVOLUTION	0.98	0.94–1.04	0.6072	0.002	0.497
PREVIOUS HOSPITALIZATION	1.76	0.99–3.14	0.0541	0.022	0.568
AGE	1.03	0.99–1.06	0.0647	0.021	0.581
WOMEN	1.24	0.70–2.21	0.4566	0.003	0.527
NYHA III-IV	2.85	1.49–5.46	0.0016	0.056	0.594
LIVE ALONE	1.24	0.62–2.45	0.5343	0.002	0.518
MEDIUM / HIGH EDUC LEVEL	0.52	0.20–1.31	0.1664	0.012	0.536
EMPLOYMENT SITUATION					
1. HOUSEWIFE	Ref:	Ref:		0.005	0.520
2. RETIRED	1.03	0.56–1.87	0.9158		
3. EMPLOYEE	0.57	0.15–2.16	0.4126		
BMI	0.99	0.94–1.03	0.6579	0.001	0.525
DIABETES	1.90	1.07–3.38	0.027	0.028	0.579
COPD	2.94	1.41–6.16	0.0041	0.046	0.574
ISCHEMIC CARDIOPATHOLOGY	0.91	0.46–1.77	0.7860	0.000	0.508
ATRIAL FIBRILLATION	1.42	0.79–2.54	0.2385	0.008	0.542
STROKE	1.74	0.86–3.51	0.1238	0.013	0.542
LVEF	1.03	1.00–1.05	0.0405	0.030	0.610
ACE / ARB	0.81	0.44–1.49	0.4988	0.003	0.522
BETA BLOQUERS	0.64	0.36–1.14	0.1295	0.013	0.555
DIURETIC	1.36	0.61–3.01	0.4515	0.003	0.521
CALCIUM CHANNEL BLOCKERS	1.29	0.69–2.43	0.4232	0.004	0.525
DIGOXIN	1.29	0.69–2.43	0.4232	0.004	0.525
PHYSICAL MLHFQ	1.03	1.01–1.06	0.0106	0.039	0.614
EMOTIONAL MLHFQ	1.08	1.03–1.13	0.0008	0.065	0.630
TOTAL MLHFQ	1.03	1.01–1.05	0.0010	0.064	0.638
UCA WHODAS SEVERE / EXTREME (%)	1.81	0.83–3.93	0.1322	0.013	0.537
GAR WHODAS SEVERE / EXTREME (%)	1.79	1.00–3.19	0.493	0.023	0.570
SCA WHODAS SEVERE / EXTREME (%)	2.76	1.33–5.75	0.0064	0.041	0.571
GAP WHODAS SEVERE / EXTREME (%)	1.72	0.75–3.95	0.2021	0.009	0.529
LAC WHODAS SEVERE / EXTREME (%)	2.61	1.46–4.67	0.0012	0.062	0.617
PSO WHODAS SEVERE / EXTREME (%)	3.26	1.75–6.06	0.0002	0.079	0.620
TOTAL WHODAS SEVERE / EXTREME (%)	2.24	1.12–4.47	0.0224	0.029	0.563

EDC: Expanded diagnostic clusters. NYHA: New York Heart Association. Body Mass Index. COPD: Chronic Obstructive Pulmonary Disease. LVEF: Left Ventricular Ejection Fraction. ACE/ARB: Ratio ACE Inhibitors /Angiotensin Receptor Blockers. MLHFQ: Minnesota Living with Heart Failure Questionnaire. WHODAS: World Health Organization Assessment Schedule. UAC: Understanding and Communication. GAR: Getting around. SCA: Self-Care. GAP: Getting along with people. LAC: Life activities. PSO: Participation in society.

The final multivariate model is presented in Tables 3 and 4 with an evaluation of performance in the development sample and in the internal validation process. The final model consists of four variables to predict the risk of hospitalization in 12 months: being in NYHA functional class III-IV, having diabetes, having COPD and the MLHFQ (quality of life questionnaire) emotional dimension score. A combination of the variables was used to determine predicted probability according to the formula (1/(1 + exp(-1*(-2.2647 + 0.8996*(NYHA_Cat2-1) + 1.1547*COPD+ 0.0697*MLHFQ_Emotional + 0.6632*DIABETES)))). The model exhibited moderate discrimination capacity, AUC 0.723, with a Hosmer-Lemeshow test p-value of 0.815 and event variance explanatory capacity of 17%. Discrimination and calibration were maintained in the internal validation process (Table 4).

**Table 3:** Final multivariate model.

Model	Coefficient	OR	95% CI	p-value
Intercept	-2.2647			0.0001
NYHA III-IV	0.8996	2.46	1.23–4.91	0.0108
DIABETES	0.6632	1.94	1.05–3.58	0.0336
COPD	1.1547	3.17	1.45–6.94	0.0039
EMOTIONAL MLHFQ	0.0697	1.07	1.02–1.12	0.0046

NYHA: New York Heart Association. Body Mass Index. COPD: Chronic Obstructive Pulmonary Disease. MLHFQ: Minnesota Living with Heart Failure Questionnaire.

**Table 4:** Performance measurements.

Model	ROC area (CI95%)	Intersections	Recalibration slope	R2N	Brier Score	Hosmer-Lemeshow Test
Final	0.723 (0.647–0.798)	0	1	0.169	0.165	0.8149
Internal validation	0.706 (0.631–0.783)	-0.0830	0.9116	0.1342	0.1734	

CI95%: Confidence Interval 95%

Based on the model developed (Table 3) to estimate the risk of hospitalization at 12 months, an easy-to-use clinical prediction rule was constructed by assigning points to the coefficients. Thus, for a patient with NYHA III-IV, with COPD, Emotional MLHFQ score of 3, who does not have DIABETES would be assigned a total of 4 points, values obtained from Table 5 (3 for NYHA III-IV, 0 for COPD, 1 for MLHFQ_Emotional and 0 for DIABETES) and a hospital admission probability of 30.24% (Table 6). Using the formula described above, a predicted probability of 23.93% is obtained.

**Table 5:** Assignment of scores in the final model.

	Categories	Reference Category	Reference value	Example subject
NYHA	Class III—IV		3	3
	Class I—II	Reference	0	X
COPD	COPD		3	X
	NO COPD	Reference	0	0
EMOTIONAL MLHFQ	< = 1	Reference	0	X
	> = 2 - < = 5		1	1
	> = 6 - < = 11		2	X
	> = 12		3	X
DIABETES	DIABETES		2	X
	NO DIABETES	Reference	0	0
Total points				4

NYHA: New York Heart Association. Body Mass Index. COPD: Chronic Obstructive Pulmonary Disease. MLHFQ: Minnesota Living with Heart Failure Questionnaire.

**Table 6:** Risk associated with point totals.

Total points	Probability
0	0.0971
1	0.1322
2	0.1776
3	0.2343
4	0.3024
5	0.3805
6	0.4653
7	0.5522
8	0.6360
9	0.7123
10	0.7782
11	0.8325

## Discussion

In our cohort of 251 HF patients, 64 were admitted for unplanned causes in a period of 12 months after inclusion in the study. 20.3% were patients who are in stage III-IV of the NYHA functional classification and with a mean LVEF of 58%. We started with a model that considers socio-demographic, clinical, functional capacity and quality of life variables as predictive factors. Four variables: NYHA functional class, diabetes, COPD and the MLHFQ emotional dimension score, were included in the final model to predict risk of hospitalization; three of these variables are part of the clinical information collected on a regular basis from the medical records of HF patients and quality of life; functional capacity is necessary given the impact of HF on these health dimensions [18]. Analysis of hospitalization over a 12-month period for any unplanned cause enables us to begin to assess the quality of hospital care and also to coordinate and provide ongoing care for these patients.

A large number of HF patient hospitalizations can be avoided [19]; identifying hospitalization risk factors can help reduce that number. The model we present has an acceptable discrimination capacity. However, its explanatory capacity of hospitalization variance is low, indicating that other relevant variables have been left out of the model. In general, models developed to predict HF patient hospitalization and mortality have been less accurate in predicting hospitalization. Hospitalization is more difficult to predict because it is an event that, in addition to being determined by the patient's clinical situation, depends on the characteristics of the healthcare system and the patient's support capacity.

In our study, 39.8% of patients have diabetes. HF and diabetes share some of the same physiopathological mechanisms [20]. The association of HF and diabetes increases the risk of hospitalization and death, compared to HF patients without diabetes [21]. Recommendations and CPGs have recently been published for the treatment of HF patients and diabetes [22,23].

COPD is a common comorbidity in HF patients in our case and in other studies; 14% of patients have COPD. In our study, COPD increased the risk of hospital admission three-fold. COPD in HF patients is associated with higher comorbidity [24], increases the number family doctor visits [25], and increases the risk of hospitalization and death in these patients [26,27]. Despite the peculiarities involved in the therapeutic management of these patients, neither the ESC’s clinical practice guidelines for HF patients nor the GOLD guidelines contemplate management of this comorbidity in depth [28].

HF Patients are seriously limited in terms of their functional capacity and quality of life, which is why experts recommend including quality of life and functional capacity as endpoints in studies with HF patients [29]. In our study, 17% of patients have a severe/extreme global disability, measured with WHODAS 2; in some domains, such as ADL, severe/extreme disability reaches 40%.

Disability has been identified as a predictor of mortality and hospital admission in elderly patients [30,31], in patients with complex ailments such as HF, these types of variables have greater predictive capacity for hospitalization than comorbidity [32]. However, the effect of disability has been less studied as a risk factor for hospital admission [31]. In our case, the limitation to perform the ADLs was a predictor of hospital admission in the univariate analysis, but the effect disappeared in the multivariate analysis.

Our study demonstrates an association between the emotional dimension of MLHFQ and hospital admission. Depression is a common mental disorder in HF patients that may explain the association found [33].

The study has limitations in terms of external validity. Since patients were recruited from family medical clinics, most were clinically stabilized HF patients with preserved ejection fraction (HFpEF) and low NYHA functional class, resulting in a unique sample.

The proposed model was validated internally but should be subjected to an external validation process in order to generalize the results.

We have not included biochemical or haematological parameters, some such as blood urea nitrogen has been shown as a relevant predictor of hospitalization in HF patients and its exclusion may have reduced the explanatory power of the model developed [4]. Patients' clinical situation determined hospitalization, but the characteristics of the health system and the hospital's admission policy were also relevant factors that were not considered in this study.

In conclusion, we have developed a simple 12-month predictive hospitalization model based on four variables: NYHA functional class, diabetes, COPD and the emotional dimension of the MLHFQ scale, that has an acceptable discriminative capacity but that should be validated externally.

## Supporting information

S1 File Database for statistical analysis is available as supporting file. (ZIP)
